# Residually Stressed Fiber Reinforced Solids: A Spectral Approach

**DOI:** 10.3390/ma13184076

**Published:** 2020-09-14

**Authors:** Mohd Halim Bin Mohd Shariff, Jose Merodio

**Affiliations:** 1 Department of Mathematics, Khalifa University of Science and Technology, Abu Dhabi 127788, UAE; mohd.shariff@ku.ac.ae; 2 Department of Continuum Mechanics and Structures, Escuela de Ingenieros de Caminos, Universidad Politecnica de Madrid, 28040 Madrid, Spain

**Keywords:** nonlinear elasticity, constitutive model, residual stress, preferred direction, spectral formulations, physical invariants, independent invariants

## Abstract

We use a spectral approach to model residually stressed elastic solids that can be applied to carbon fiber reinforced solids with a preferred direction; since the spectral formulation is more general than the classical-invariant formulation, it facilitates the search for an adequate constitutive equation for these solids. The constitutive equation is governed by spectral invariants, where each of them has a direct meaning, and are functions of the preferred direction, the residual stress tensor and the right stretch tensor. Invariants that have a transparent interpretation are useful in assisting the construction of a stringent experiment to seek a specific form of strain energy function. A separable nonlinear (finite strain) strain energy function containing single-variable functions is postulated and the associated infinitesimal strain energy function is straightforwardly obtained from its finite strain counterpart. We prove that only 11 invariants are independent. Some illustrative boundary value calculations are given. The proposed strain energy function can be simply transformed to admit the mechanical influence of compressed fibers to be partially or fully excluded.

## 1. Introduction

The presence of residual stresses in solids has been the essence of numerous publications [1,2,3]. There is a considerable interest in the mechanical behaviour of residually stressed materials in recent years and attempts to comprehend the mechanical behaviour of residual stresses on solid materials can be found in the literature [2,4,5,6]. A review on the presence of residual stress, for example, in fiber reinforced composite materials can be found in [7]. In this paper, we focus on the modelling of the mechanical anisotropic response of a residually stressed fiber material with a preferred direction (RSPD) based on the spectral method (method that used the eigenvalues and eigenvectors of tensors) developed recently in the literature [8,9,10,11,12,13,14,15,16]. Applications can be found, for example, in the mathematical modelling of the mechanical behaviour of carbon fibre reinforced solids and other types of RSPD, like soft biological tissue. We note that, before the recent applications of spectral formulation in anisotropic solids, most anisotropic models used traditional classical invariants [17] (or their variants), where majority of them do not have a direct interpretation, to describe their strain energy functions (see, for example, [1,18]). However, the proposed strain energy function in this paper uses spectral invariants, each has a transparent meaning that is convenient for experimental design [19]. A discussion of the advantages of spectral invariants over classical invariants is given in [20].

In this communication, our objective is to develop a novel strain energy function using spectral invariants that contains only single-variable functions. These strain energy types are experimentally attractive [19] and have been fortunate in modelling non-elastic and elastic solids [8,9,10,11,12,13,14,15,16,20,21]. It is important to note that knowing the number of independent invariants facilitates a stringent development of a strain energy via an experiment [22], and in our spectral analysis, we derive that only 11 independent spectral invariants are required in the constitutive equation. Up to our present knowledge, we believe that, modelling RSPDs using 11 independent spectral invariants is novel. We must emphasize that, to the best of our knowledge, since we are not able to find an appropriate residual stress experiment data of materials with a preferred direction, this paper focuses on the development of a rigourous theoretical spectral constitutive equation based on a systematic and rigorous use of the restrictions imposed by thermodynamics, the derivation and use of the representation formulae, a consequence of the rigorous definitions of the different material behaviors and of the concept of material symmetry, and a priori restrictions that are required by well posed mathematical models.

The basic kinematic deforming body equations and basic properties of residual stresses are given in Section 2. We discuss spectral formulations in Section 3. In Section 4, a spectral strain energy function in the absence of residual stress is proposed and, in Appendix C, based on the wok of Shariff [14], its extension to fully or partially exclude the mechanical influence of compressed fibers is given. In Section 5, a prototype strain energy function that contains single-variable functions is proposed. This prototype function is used in Section 6 to study some boundary value problems. Conclusions are given in Section 7.

## 2. Main Equations

### 2.1. Basic Concepts

Unless stated otherwise, all subscripts *i*, *j* and *k* take the values 1–3 and the summation convention is not used. We only discuss quasi-static deformations of incompressible solids with negligible body forces. The right Cauchy-Green tensor is $C=F^{T}F=U^{2}$, where $F=\frac{\partial x}{\partial X}$ is the deformation tensor, *U* is the right stretch tensor, and *X* and *x* denote the position vectors of a solid body particle, respectively, in the reference and current configurations. Since the material is incompressible, det(F)=1, where det denotes the determinant of a tensor. More details about the kinematics of deforming bodies and the equation of motion can be found, for example, in Ref. [23].

### 2.2. Residual Stress

Details on the definition of a residual stress can be found in Merodio et al. [6]. Briefly, we postulate the existence of an equilibrium stress field with zero traction on the surface of a body in a reference configuration $B_{r}$; the term residual stress $S_{R}$ is often used for this equilibrium stress. Hence,

(1) $$\begin{array}{c} \mathrm{Div}S_{R}=0\;\mathrm{in}\;B_{r} \end{array}$$

with the condition on the boundary

(2) $$\begin{array}{c} S_{R}N=0\;\mathrm{on}\;\partial B_{r}\;, \end{array}$$

where *N* is unit outward normal to $\partial B_{r}$, the boundary of $B_{r}$ and Div is the divergence operator with respect to *X*. In account of (1) and (2), the residual stress has the mean value

(3) $$\begin{array}{c} \int_{B_{r}}S_{R}\;dV=0\;, \end{array}$$

and it is non-homogeneous.

## 3. Spectral Representation

Following the work of Shariff and Merodio [20], the strain energy Ω is postulated as follows:

(4) $$\begin{array}{c} \Omega =W_{\left(a\right)}\left(U,S_{R},A\right)\;,\;A=a⊗a\;, \end{array}$$

where ⊗ denotes the dyadic product. For an incompressible material, the Cauchy stress *S* for an incompressible solid is given by

(5) $$\begin{array}{c} S=2F\frac{\partial \Omega }{\partial C}F^{T}-pI\;, \end{array}$$

where the Lagrange multiplier *p* is associated with the constraint detF=1 and *I* is the identity tensor. In view of the description of $S_{R}$ in Section 2.2, the constitutive Equation (5) at the reference configuration (F=I) must satisfy the relation

(6) $$\begin{array}{c} S_{R}=2\frac{\partial W_{\left(a\right)}}{\partial C}\left(I,S_{R},A\right)-p_{0}I\;, \end{array}$$

where the Lagrange multiplier $p_{0}$ is the value of *p* at the reference configuration.

We note that the right stretch tensor

(7) $$\begin{array}{c} U=\sum_{i=1}^{3}\lambda_{i}u_{i}⊗u_{i}\;, \end{array}$$

where $\lambda_{i}$ (principal stretches) and $u_{i}$ are eigenvalues and eigenvectors and of *U*, respectively. With respect to the basis $\left\{u_{1},u_{2},u_{3}\right\}$, we express Ω in terms of the 12 components

(8) $$\begin{array}{c} \lambda_{i}\;,\;s_{ij}=u_{i}\cdot S_{R}u_{j}\;,\;a_{i}=a\cdot u_{i}\;, \end{array}$$

where · is the dot product between two vectors. The unit vector *a* implies

(9) $$\begin{array}{c} a_{3}^{2}=1-a_{1}^{2}-a_{2}^{2}\; \end{array}$$

which proves that only 11 of the 12 components are independent. Since, for all rotation tensor *Q*,

(10) $$\begin{array}{c} s_{ij}=u_{i}\cdot S_{R}u_{j}=Qu_{i}\cdot QS_{R}Q^{T}Qu_{j}\;,\;a_{i}=a\cdot u_{i}=Qa\cdot Qu_{i}\;, \end{array}$$

it is clear that, with respect to *Q*, $s_{ij}$ and $a_{i}$ are invariants. We emphasize that although $a_{i}$ are invariants, they are not invariants for the tensor set $ST=\{U,S_{R},A\}$. The invariants $\lambda_{i},s_{ij}$ and $a_{i}^{2}$, for example, are invariants for the tensor set ST.

In Appendix A, relations between classical invariants [17] are given, which strengthen our claim that at most 11 invariants are independent. We emphasize that, unlike the spectral invariants given in (8), most of 18 classical invariants in the minimal integrity basis do not have a clear physical meaning.

According to Shariff [13], the strain energy function must satisfy the *P*-property [13]. To facilitate the construction of the *P*-property, the following six independent spectral invariants

(11) $$\begin{array}{c} \zeta_{i}=s_{ii}=u_{i}\cdot S_{R}u_{i}\;,\;{\bar{\zeta }}_{i}=u_{i}\cdot S_{R}^{2}u_{i}\; \end{array}$$

are used rather than the invariants $s_{ij}$. Hence, the strain energy Ω can be expressed as

(12) $$\begin{array}{c} \Omega =W_{\left(b\right)}\left(\lambda_{i},a_{i},\zeta_{i},{\bar{\zeta }}_{i}\right)\;. \end{array}$$

The Lagrangean components [24] of $\frac{\partial \Omega }{\partial C}$ with respect to the basis $\left\{u_{1},u_{2},u_{3}\right\}$ are required in our analysis and they are [19]:

(13) $$\begin{array}{c} {\left(\frac{\partial \Omega }{\partial C}\right)}_{ii}=\frac{1}{2\lambda_{i}}\frac{\partial W_{\left(b\right)}}{\partial \lambda_{i}}\;\left(i\;\mathrm{not}\;\mathrm{summed}\right) \end{array}$$

with shear components

(14) $$\begin{array}{ccc} {\left(\frac{\partial \Omega }{\partial C}\right)}_{ij} & = & \frac{\frac{\partial W_{\left(b\right)}}{\partial u_{i}}\cdot u_{j}-\frac{\partial W_{\left(b\right)}}{\partial u_{j}}\cdot u_{i}}{2(\lambda_{i}^{2}-\lambda_{j}^{2})},\;i\neq j\;. \end{array}$$

The Eulerian components [24] of Cauchy stress *S* with respect to the basis $\left\{v_{1},v_{2},v_{3}\right\}$, where $v_{i}\;=\;FU^{-1}u_{i}$ are

(15) $$\begin{array}{ccc} \tau_{ii} & = & \lambda_{i}\frac{\partial W_{\left(b\right)}}{\partial \lambda_{i}}-p\;,\\ \tau_{ij} & = & 2\lambda_{i}\lambda_{j}{\left(\frac{\partial \Omega }{\partial C}\right)}_{ij}\;,\;i\neq j\;. \end{array}$$

## 4. Transversely Isotropic Elastic Solid without Residual Stress

Prior to constructing a prototype strain energy function for RSPDs, we initially discuss the spectral constitutive equation for transversely isotropic elastic solids in the absence of residual stress, see for example the work of Shariff [14]. In this section, we construct a general nonlinear (finite strain) spectral strain energy function for solids with a preferred direction. We must emphasize that a finite-strain energy function should be consistent with its infinitesimal-strain counterpart (see Ref. [20] for details). The “infinitesimal strain” approach has two advantages: (a) the nonlinear strain energy function contains separable single-variable functions [14], which are easier to analyse than multivariable functions and (b) the strain energy function can be easily amended to fully or partially exclude the mechanical influence of compressed fibers (see Appendix C).

### 4.1. Infinitesimal Strain

In infinitesimal deformations, the most general quadratic form strain energy function for an incompressible transversely isotropic material has the expression [14]

(16) $$\begin{array}{c} W_{\left(T\right)}=\mu_{T}K_{1}+2\mu_{1}K_{2}+\frac{\beta }{2}K_{3}^{2}\;, \end{array}$$

where

(17) $$\begin{array}{c} K_{1}=\sum_{i=1}^{3}\nu_{i}^{2}=\mathrm{tr}\;E^{2}\;\;,\;K_{2}=\sum_{i=1}^{3}{\bar{a}}_{i}^{2}\nu_{i}^{2}=a\cdot E^{2}a,\;K_{3}=\sum_{i=1}^{3}{\bar{a}}_{i}^{2}\nu_{i}=a\cdot Ea\;, \end{array}$$

(18) $$\begin{array}{c} \mu_{1}=\mu_{L}-\mu_{T}\;, \end{array}$$

$\mu_{L}$ and $\mu_{T}$ are shear moduli, β is a ground-state constant that is related to other elastic constants, which have more direct physical interpretations, ${\bar{a}}_{i}=e_{i}\cdot a$, $e_{i}$ is an eigenvector of the infinitesimal strain *E* and $\nu_{i}$ is an eigenvalue of *E*.

### 4.2. Finite Strain

With the aid of the infinitesimal form (16), we postulate a finite-strain energy function

(19) $$\begin{array}{c} W_{\left(T\right)}=\sum_{i=1}^{3}\left[\mu_{T}q_{1}\left(\lambda_{i}\right)+\mu_{1}\alpha_{i}q_{2}\left(\lambda_{i}\right)\right]+\frac{\beta }{2}{\left(\sum_{i=1}^{3}\alpha_{i}q_{3}\left(\lambda_{i}\right)\right)}^{2}\;, \end{array}$$

where $\alpha_{i}=a_{i}^{2}$. The restrictions [14]

(20) $$\begin{array}{c} q_{s}\left(1\right)=0\;,\;s=1,2,3\;,\;q_{1}^{\prime }\left(1\right)=q_{2}^{\prime }\left(1\right)=0\;,\;q_{1}^{\prime \prime }\left(1\right)=q_{2}^{\prime \prime }\left(1\right)=2\;,\;q_{3}^{\prime }\left(1\right)=1\; \end{array}$$

are required so that the finite-strain energy function is consistent with infinitesimal strain theory. In view of detF=1, we could also impose $q_{1}^{\prime }\left(1\right)=q_{1}^{\prime \prime }\left(1\right)=1$.

For $|\lambda_{i}-1|<<1$, the value of the nonlinear strain energy function is close to the value of the corresponding infinitesimal strain energy function, and for this range of strains the strain energy function can be approximated by letting

(21) $$\begin{array}{c} q_{1}\left(\lambda_{i}\right)=q_{2}\left(\lambda_{i}\right)={\left(\lambda_{i}-1\right)}^{2}\;,\;q_{3}=\lambda_{i}-1\;. \end{array}$$

It is clear that $q_{1},q_{2}$ and $q_{3}$ satisfy the properties in (20) and the property

(22) $$\begin{array}{c} q_{1}^{\prime \prime }>0\;,\;q_{2}^{\prime \prime }>0\;. \end{array}$$

In view of (21), $q_{1}^{\prime },q_{2}^{\prime }$ and $q_{3}$ are monotonically increasing functions with $q_{1}^{\prime },q_{2}^{\prime }$ and $q_{3}$ negative and positive for $\lambda_{i}<1$ and $\lambda_{i}>1$, respectively. From a continuity point of view, we shall assume that the functions $q_{1},q_{2}$ and $q_{3}$ have the above properties for all ranges of $\lambda_{i}$. The connection of $q_{1},q_{2}$ and $q_{3}$ to the generalized Lagrangean strain tensor is given in Appendix B. The concepts of polyconvexity, strong ellipticity condition at the current configuration, convexity and stability can be used to put restrictions on the functions $q_{1}$, $q_{2}$ and $q_{3}$. However, the application of these concepts to our constitutive equation is beyond the scope of this paper. The amended strain energy function proposed in this Section to model the ramification of compressed fibers is given in Appendix C.

The general single-variable functions that depend on a principal stretch $\lambda_{i}$, appearing in $W_{\left(T\right)}$, facilitate the construction of a specific form of strain energy function from experimental data. We note that, recently, separable single-variable strain energy functions have been used to model both elastic and non-elastic solids [8,9,10,11,12,13,14]. We note in passing that, it is shown in Shariff [14], the constitutive Equation (19) has successfully predicted the mechanical behaviour of soft tissues. Below, we give an example, where our theory is compared with the experimental data of [25]. We strongly emphasize that the following specific forms given below to fit the experimental data of [25] is just a preliminary exercise; better functional forms could be obtained for $q_{1},q_{2}$ and $q_{3}$ but it is not the intention of this paper to do so.

For rubberlike materials, the specific forms are used in Shariff [9]:

(23) $$\begin{array}{c} q\left(x\right)=q_{1}\left(x\right)=q_{2}\left(x\right)=2\left(xln\left(x\right)-x+1\right)+d_{0}\left(-e^{1-x}+\frac{x^{2}-4x+5}{2}\right)+d_{1}\left(e^{x-1}-\frac{x^{2}+1}{2}\right)\; \end{array}$$

and

(24) $$\begin{array}{c} q_{3}\left(x\right)=ln\left(x\right)\;. \end{array}$$

**Figure 1:**
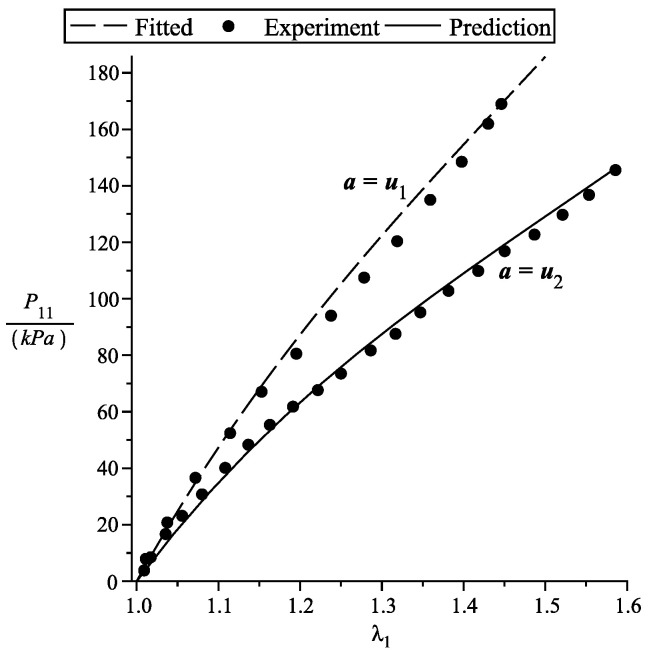
First Piola–Kirchhoff stress vs stretch. Ciarletta et al. [25] fiber reinforced simple tension experiment. $\mu_{T}=120kPa$, $\mu_{L}=160kPa$, β=0, $d_{0}=-3$, $d_{1}=2$. The experiment data is adapted from [25].

We compare our theory with the uniaxial experiment of Ciarletta et al. [25] on fiber reinforced rubber, where the experimental characterization is performed using a uniaxial testing device with optical measures of the deformation, using two different reinforcing materials on a ground rubber matrix. In The uniaxial stretch is in the $u_{1}$ direction. The non-zero axial First Piola–Kirchhoff stress component is

(25) $$\begin{array}{c} P_{11}=\left(\mu_{T}+2\mu_{1}\right)q^{\prime }\left(\lambda_{1}\right)+\beta q_{3}\left(\lambda_{1}\right)q_{3}^{\prime }\left(\lambda_{1}\right)-\mu_{T}\frac{\lambda_{3}q^{\prime }\left(\lambda_{3}\right)}{\lambda_{1}}\; \end{array}$$

for the case when $a=u_{1}$ and in this case $\lambda_{3}=\frac{1}{\sqrt{\lambda_{1}}}$. In Figure 1 we curve fit the $a=u_{1}$ data (visually) since we know that $\lambda_{3}=\frac{1}{\sqrt{\lambda_{1}}}$.

However, we cannot curve fit for the case $a=u_{2}$, since the values of $\lambda_{3}$ are unknown. In view of this, we have no choice but to predict the experimental data using the relation

(26) $$\begin{array}{c} P_{11}=\mu_{T}\left(q^{\prime }\left(\lambda_{1}\right)-\frac{\lambda_{3}q^{\prime }\left(\lambda_{3}\right)}{\lambda_{1}}\right)\;, \end{array}$$

where the dependence of $\lambda_{3}$ on $\lambda_{1}$ is obtained from solving the First Piola–Kirchhoff component equations $P_{22}=P_{33}=0$ and $\lambda_{1}\lambda_{3}\lambda_{3}=1$. It is clear from Figure 1, that we are able to predict and fit using the above specific forms.

## 5. Strain Energy for RSPD

Due to the lack of experimental data in the literature, we are impelled to postulate a simple prototype

(27) $$\begin{array}{c} \Omega =W_{\left(T\right)}+\sum_{i=1}^{3}\zeta_{i}r\left(\lambda_{i}\right)=\tilde{\Omega }\left(\lambda_{i},\alpha_{i},\zeta_{i}\right)\; \end{array}$$

based on the work of Shariff et al. [26,27], where

(28) $$\begin{array}{c} r\left(1\right)=0\;,\;r^{\prime }\left(1\right)=1\;. \end{array}$$

An example of $r(\lambda_{i})$ are $r\left(\lambda_{i}\right)=\lambda_{i}-1$ and $r\left(\lambda_{i}\right)=\ln\lambda_{i}$. In this paper, we use $r\left(\lambda_{i}\right)=\lambda_{i}-1$ for the results given in Section 6.

We could, of course, propose a more complex specific form (see, for example, Shariff et al. [26]), but in this paper, for simplicity, we only consider the specific form given in (27).

We note that for the case of $q_{1}^{\prime }\left(1\right)=q_{1}^{\prime \prime }\left(1\right)=1$ Equation (6) becomes

(29) $$\begin{array}{c} S_{R}=\left(\mu_{T}-p_{0}\right)I+S_{R}\;, \end{array}$$

which implies

(30) $$\begin{array}{c} \mu_{T}=p_{0}\;, \end{array}$$

and for $q_{1}^{\prime }\left(1\right)=0\;,q_{1}^{\prime \prime }\left(1\right)=2$, Equation (6) takes the form

(31) $$\begin{array}{c} S_{R}=-p_{0}I+S_{R}\;, \end{array}$$

and it follows that $p_{0}=0$.

The values of the residual stress and the ground state constants are restricted via the condition of strong ellipticity at the reference configuration (see Appendix D).

The spectral components of $\frac{\partial \Omega }{\partial C}$ for the strain energy function (27) takes the form

(32) $$\begin{array}{c} {\left(\frac{\partial \Omega }{\partial C}\right)}_{ii}=\frac{1}{2\lambda_{i}}\frac{\partial \tilde{\Omega }}{\partial \lambda_{i}}\;\left(i\;\mathrm{not}\;\mathrm{summed}\right) \end{array}$$

with shear components

(33) $$\begin{array}{ccc} {\left(\frac{\partial \Omega }{\partial C}\right)}_{ij} & = & \frac{1}{\left(\lambda_{i}^{2}-\lambda_{j}^{2}\right)}\left\{\left(\frac{\partial \tilde{\Omega }}{\partial \zeta_{i}}-\frac{\partial \tilde{\Omega }}{\partial \zeta_{j}}\right)u_{i}\cdot S_{R}u_{j}+\left(\frac{\partial \tilde{\Omega }}{\partial \alpha_{i}}-\frac{\partial \tilde{\Omega }}{\partial \alpha_{j}}\right)u_{i}\cdot Au_{j}\right\}\;. \end{array}$$

## 6. Boundary Value Problems

In this section we give results for a simple tension deformation of a cylinder and, expansion and contraction of a hollow sphere, which could be useful from the numerical and/or experimental point of view. The boundary value problems discussed below are for any types of RSPD and, since we are not able to find an appropriate residual stress experiment data of materials with a preferred direction, only theoretical residual stress fields are discussed in this section, which may (or may not) represent “real” residual stress fields found in RSPDs.

### 6.1. Residual Stress: Cylinder

The study of non-residually stressed fibre reinforced solids on a cylindrical configuration is a subject of numerous publications, see for example, Ref. [28]. The cylindrical residual stress results obtained in this paper may be used to study the mechanical influence of residual stress on cylindrical problems. To facilitate our study, we need to assume a residual stress field in the reference configuration, which is described by

(34) $$R_{a}\leq R\leq R_{b},\;0\leq \Theta \leq 2\pi ,\;0\leq Z\leq L,$$

where *R*, Θ and *Z* are reference cylindrical polar coordinates.

We consider a residual stress [6] of the form

(35) $$S_{R}=s_{1}\left(R\right)E_{R}⊗E_{R}+s_{2}\left(R\right)E_{\Theta }⊗E_{\Theta },,$$

where $E_{R}$ and $E_{\Theta }$ are cylindrical polar coordinate vectors in the reference configuration.

Satisfaction of the equilibrium equation and boundary conditions, require

(36) $$R\frac{ds_{1}}{dR}=s_{2}-s_{1},$$

and

(37) $$s_{1}\left(R_{a}\right)=0,\;s_{1}\left(R_{b}\right)=0\;.$$

A simple example of $s_{1}$ is

(38) $$s_{1}=\bar{\alpha }\left(R-R_{a}\right)\left(R-R_{b}\right)\;,$$

where $\bar{\alpha }$ is a constant. We use (38) in the following section.

### 6.2. Uniform Extension of a Cylinder

In this Section, all tensor and vector components are cylindrical polar components.

We consider a uniform extension of a cylinder with $R_{a}=0$ described by

(39) $$\begin{array}{c} r=\frac{1}{\sqrt{\lambda_{z}}}R\;,\;\theta =\Theta \;,\;z=\lambda_{z}Z\;, \end{array}$$

where (r,θ,z) is the polar coordinate in the deformed configuration. It follows that

(40) $$\begin{array}{c} F\equiv \mathrm{diag}(\frac{1}{\sqrt{\lambda_{z}}},\frac{1}{\sqrt{\lambda_{z}}},\lambda_{z})\;. \end{array}$$

Therefore, $\lambda_{1}=\lambda_{r}=\frac{1}{\sqrt{\lambda_{z}}},\lambda_{2}=\lambda_{r}=\frac{1}{\sqrt{\lambda_{z}}}\;,\lambda_{3}=\lambda_{z}$, $u_{1}=E_{R},u_{2}=E_{\Theta }$ and $u_{3}=E_{Z}$. Here, for simplicity, we let $a=E_{R}$ and hence $\alpha_{1}=1,\alpha_{2}=\alpha_{3}=0$.

The non-zero Cauchy stress components are $\sigma_{rr}$ (radial stress), $\sigma_{\theta \theta }$ (hoop stress) and $\sigma_{zz}$ (axial stress), where

(41) $$\begin{array}{c} \sigma_{rr}=\lambda_{r}\frac{\partial \tilde{\Omega }}{\partial \lambda_{1}}-p\;,\;\sigma_{\theta \theta }=\lambda_{r}\frac{\partial \tilde{\Omega }}{\partial \lambda_{2}}-p\;,\;\sigma_{zz}=\lambda_{z}\frac{\partial \tilde{\Omega }}{\partial \lambda_{3}}-p\;. \end{array}$$

$\tilde{\Omega }$ depends on *r* (or in view of (39), equivalently on *R*) and the Cauchy stress is inhomogeneous. It is clear that we have zero shear stresses and the equilibrium equation reduces to

(42) $$\begin{array}{c} r\frac{d\sigma_{rr}}{dr}=\sigma_{\theta \theta }-\sigma_{rr}\;, \end{array}$$

which can be integrated to give

(43) $$\begin{array}{c} \sigma_{rr}=\int_{b}^{r}\left(\lambda_{r}\frac{\partial \tilde{\Omega }}{\partial \lambda_{2}}-\lambda_{r}\frac{\partial \tilde{\Omega }}{\partial \lambda_{1}}\right)\frac{dr}{r}\;, \end{array}$$

where $b=\frac{R_{b}}{\sqrt{\lambda_{z}}}$. The axial stress is given by

(44) $$\begin{array}{c} \sigma_{zz}=\lambda_{z}\frac{\partial \tilde{\Omega }}{\partial \lambda_{3}}-\lambda_{r}\frac{\partial \tilde{\Omega }}{\partial \lambda_{1}}+\sigma_{rr}\;. \end{array}$$

**Figure 2:**
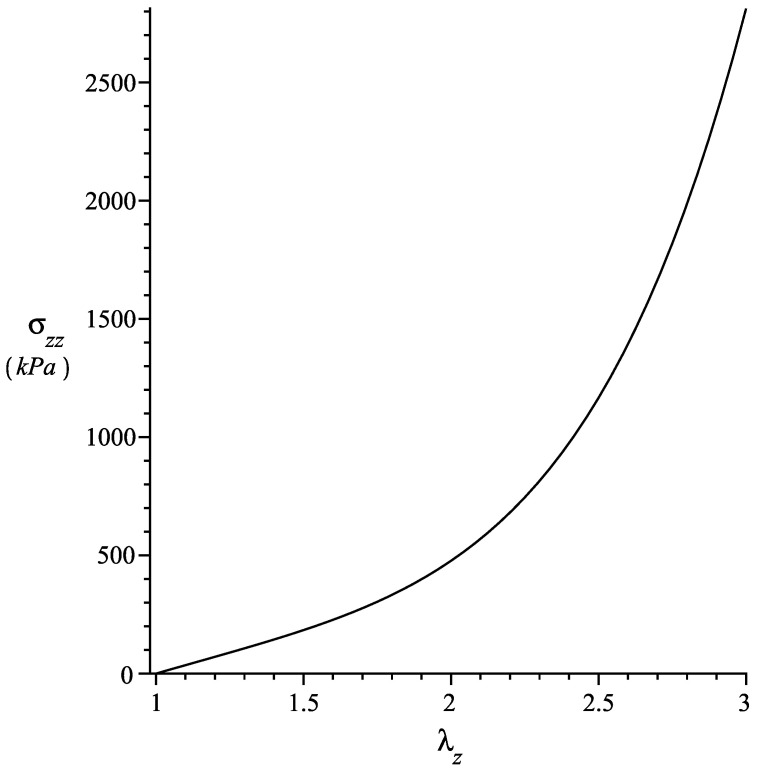
Axial stress $\sigma_{zz}$ vs axial stretch $\lambda_{z}$. $\mu_{T}=120$ kPa, β=0, $d_{0}=-3$, $d_{1}=2$.

**Figure 3:**
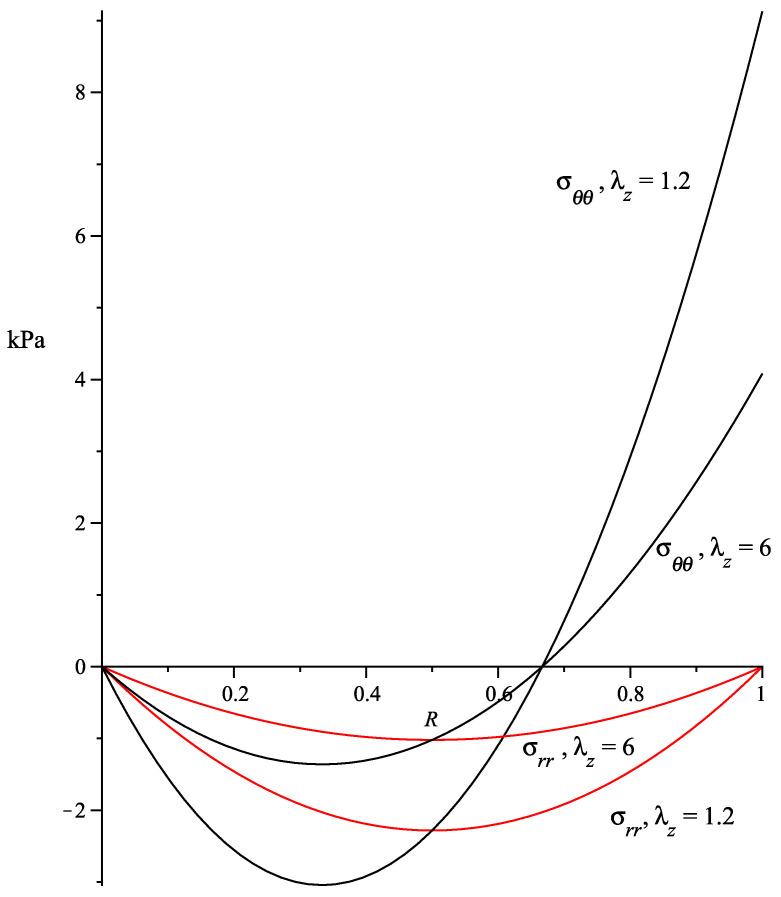
$\sigma_{rr}$ and $\sigma_{\theta \theta }$ stress fields along the radius *R*. $\bar{\alpha }=10\frac{\mathrm{kPa}}{m^{2}}$.

For illustration we use the (23), (24) and (A17) for $\tilde{\Omega }$ and we simply have

(45) $$\begin{array}{c} \sigma_{zz}=\mu_{T}\left[\lambda_{z}q^{\prime }\left(\lambda_{z}\right)-\frac{1}{\sqrt{\lambda_{z}}}q^{\prime }\left(\frac{1}{\sqrt{\lambda_{z}}}\right)\right]\;, \end{array}$$

(46) $$\begin{array}{c} \sigma_{rr}=\frac{1}{\sqrt{\lambda_{z}}}s_{1}\left(R\right)\; \end{array}$$

and

(47) $$\begin{array}{c} \sigma_{\theta \theta }=\frac{1}{\sqrt{\lambda_{z}}}s_{2}\left(R\right)\;, \end{array}$$

where, since $I_{4}\leq 1$, we have considered $\mu_{1}=\beta =0$. The above indicates that $\sigma_{zz}$ is constant and does not depend on the residual stress. On the other hand, $\sigma_{rr}$ and $\sigma_{\theta \theta }$ are functions of the residual stress only; their absolute values decrease as $\lambda_{z}$ increases. The axial Cauchy stress $\sigma_{zz}$ vs $\lambda_{z}$ is shown in Figure 2 and, in Figure 3, we plot the curves of $\sigma_{rr}$ and $\sigma_{\theta \theta }$ for $\bar{\alpha }=10\frac{kPa}{m^{2}}$.

### 6.3. Spherically Symmetric Deformation of a Spherical Shell

The study of spherical shell in this section could be useful, for example, to enhance the study of cavity formation in a sphere under uniform radial tensile dead-load with the fiber in the radial direction [29]. Here, we consider a spherical shell with thick-walled having the reference geometry

(48) $$\begin{array}{c} R_{a}\leq R\leq R_{b}\;,\;0\leq \Theta \leq \pi \;,\;0\leq \Phi \leq 2\pi \;, \end{array}$$

where (R,Θ,Φ) is the spherical polar coordinate for the undeformed configuration. The geometry of the current deformation is described by

(49) $$\begin{array}{c} a\leq r\leq b\;,\;\theta =\Theta \;,\;\phi =\Phi \;, \end{array}$$

where (r,θ,ϕ) is the spherical polar coordinate for the current configuration.

We consider a deformation defined by

(50) $$\begin{array}{c} r=Rf(R)\;. \end{array}$$

In the spherical polar coordinate system, the deformation gradient is

(51) $$\begin{array}{c} F\equiv \left(\begin{array}{ccc} 1+Rf^{\prime }\left(R\right) & 0 & 0\\ 0 & f(R) & \\ 0 & 0 & f(R) \end{array}\right)\;. \end{array}$$

Due to the incompressibility condition we have

(52) $$\begin{array}{c} f\left(R\right)={\left(1+\frac{a^{3}-R_{a}^{3}}{R^{3}}\right)}^{\frac{1}{3}}=\frac{r}{R}\;. \end{array}$$

The principal stretches are

(53) $$\begin{array}{c} \lambda_{1}=\frac{1}{\lambda^{2}}\;,\;\lambda_{2}=\lambda_{3}=\lambda =\frac{r}{R}\; \end{array}$$

and, we have, $u_{1}=E_{R}$, $u_{2}=E_{\Theta }$ and $u_{3}=E_{\Phi }$, where $\left\{E_{R},E_{\Theta },E_{\Phi }\right\}$ is the spherical polar coordinate basis for the reference configuration.

Here, we use the residual stress

(54) $$\begin{array}{c} S_{R}=s_{1}\left(R\right)E_{R}⊗E_{R}+s_{2}\left(R\right)E_{\Theta }⊗E_{\Theta }+s_{2}\left(R\right)E_{\Phi }⊗E_{\Phi }\;, \end{array}$$

where

(55) $$\begin{array}{c} s_{1}\left(R\right)=\kappa \left(R-R_{a}\right)\left(R-R_{b}\right)\; \end{array}$$

and κ has the dimension kPa/m^2^. The equilibrium equation requires

(56) $$\begin{array}{c} s_{2}\left(R\right)=\frac{1}{2R}\frac{d(R^{2}s_{1}\left(R\right))}{dR}\;. \end{array}$$

The free stress surface (2) is clearly satisfied.

We only discuss, for simplicity, the case when $a=E_{R}$ and, we simply have, for the non-stretch invariants

(57) $$\begin{array}{c} \alpha_{1}=1\;,\;\alpha_{2}=\alpha_{3}=0\;,\;\zeta_{1}=s_{1}\;,\;\zeta_{2}=\zeta_{3}=s_{2}\;. \end{array}$$

The non-zero Cauchy stress components (with respect to the spherical coordinate system) are $\tau_{rr}$, $\tau_{\theta \theta }$ and $\tau_{\phi \phi }$.

Since $\zeta_{2}=\zeta_{3}$, from (15), we have,

(58) $$\begin{array}{c} \tau_{\theta \theta }=\tau_{\phi \phi }\;. \end{array}$$

Hence, the equilibrium equation reduces to

(59) $$\begin{array}{c} \frac{d\tau_{rr}}{dr}+\frac{2}{r}\left(\tau_{rr}-\tau_{\theta \theta }\right)=0\;. \end{array}$$

Integrating (59) and taking account that if we assume that radial stress vanishes at r=a, we get

(60) $$\begin{array}{c} \tau_{rr}=\int_{a}^{r}\frac{2}{r}\left(\lambda \frac{\partial \tilde{\Omega }}{\partial \lambda_{2}}-\frac{1}{\lambda^{2}}\frac{\partial \tilde{\Omega }}{\partial \lambda_{1}}\right)\;dr\;. \end{array}$$

**Figure 4:**
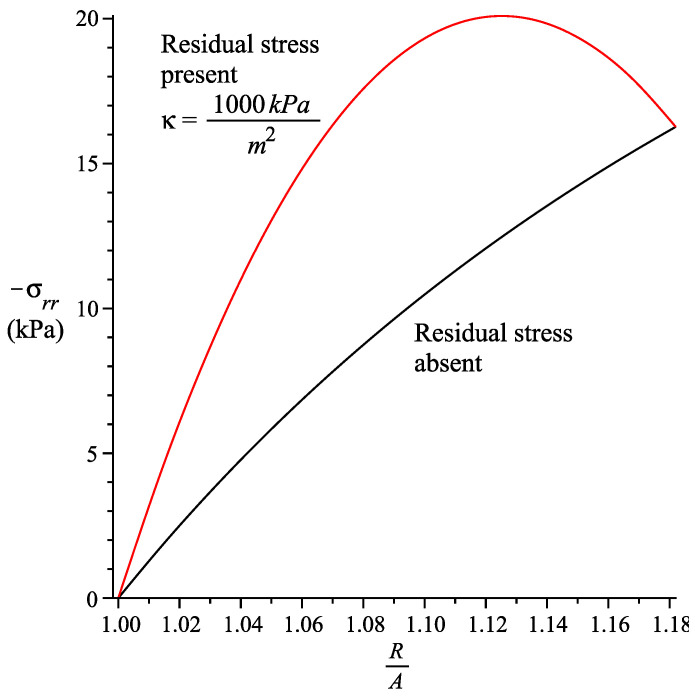
Radial stress for spherically symmetric deformation of a spherical shell. $\frac{a}{R_{a}}=0.95$, $\frac{R_{b}}{R_{a}}=1.182$, $\frac{b}{R_{b}}=0.9703$. fiber is in tension $\frac{b-a}{R_{b}-R_{a}}=1.082$.

**Figure 5:**
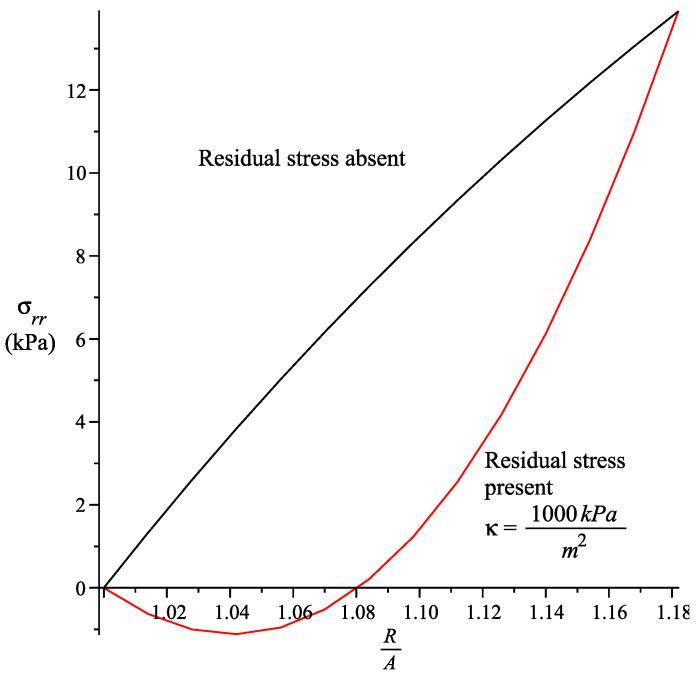
Radial stress for spherically symmetric deformation of a spherical shell. $\frac{a}{R_{a}}=1.1$, $\frac{R_{b}}{R_{a}}=1.182$, $\frac{b}{R_{b}}=0.942$. fiber is in compression $\frac{b-a}{R_{b}-R_{a}}=0.858$.

The radial stress is depicted in Figure 4 for $a/R_{a}=0.95$ (the sphere is moved inwards). We only consider $R_{b}/R_{a}=1.182$. While the fiber is in tension with $\frac{b-a}{R_{b}-R_{a}}=1.082$, a negative radial stress is obtained; taking note that $\frac{b}{R_{b}}=0.970$. Figure 4 indicates that the absolute value of the radial stress $\tau_{rr}$ increases in the present of a residual stress. However, when the sphere is moved outwards (see Figure 5), we have, $a/R_{a}=1.1$, $\frac{b}{R_{b}}=1.062787231$, $\frac{b-a}{R_{b}-R_{a}}=0.8583214670$ and hence the fiber is in compression. We note that the radial stress is negative in some segments due to the presence of residual stress. In this section the values $\mu_{1}=\beta =0$ are used for the graphs.

## 7. Conclusions

In this communication, we propose a novel separable spectral strain energy function for RSPDs which contains single-variable spectral-invariant functions. The use of spectral invariants is the state of the art in modelling RSPDs and has the advantage that each of the spectral invariants has a clear physical interpretation, which is useful in aiding the design of experiments. The spectral invariants depend on the right stretch tensor, the preferred direction and the residual stress tensor. Our approach ensures that the strain energy function for infinitesimal strain can be simply obtained from its finite strain counterpart and vice-versa. The analysis of the boundary value problems given in Section 6, highlights the simplicity of the spectral approach. Modelling full or partial exclusion of compressed fibers is simply done by amending the strain energy function. In future, we require experimental data to compare our theory with different types of RSPDs. Since classical invariants can be explicitly expressed in terms of spectral invariants but not vice versa, spectral formulations are, hence, more general. 

## Appendix A

It is obvious that since all the classical invariants given below can be expressed in terms of the spectral invariants, only 11 of them are independent. To strengthen our claim, in this Appendix, we construct relations between classical invariants to prove that only 11 of the classical invariants are independent. The construction of relations between classical invariants requires the invariants

(A1) $$\begin{array}{ccc} I_{1} & = & \mathrm{tr}\;C=\sum_{i=1}^{3}\lambda_{i}^{2}\;,\\ I_{2} & = & \frac{1}{2}\left({\left(\mathrm{tr}\;C\right)}^{2}-\mathrm{tr}\;C^{2}\right)=\lambda_{1}^{2}\lambda_{2}^{2}+\lambda_{1}^{2}\lambda_{3}^{2}+\lambda_{2}^{2}\lambda_{3}^{2}\;,\\ I_{3} & = & \det\left(C\right)=\lambda_{1}^{2}\lambda_{2}^{2}\lambda_{3}^{2}\;. \end{array}$$

It is commonly known that the above relations are independent and since the eigenvalues $\lambda_{i}$ are independent, there are no relations between the classical invariants and hence the three classical invariants are independent. The eigenvalues $\lambda_{i}$ we can be explicitly expressed in terms of the classical invariants [30], i.e.,

(A2) $$\begin{array}{c} \lambda_{i}^{2}=\frac{1}{3}\left\{I_{1}+2\sqrt{I_{1}^{2}-3I_{2}}\cos\frac{1}{3}\left[\theta +2\pi \left(i-1\right)\right]\right\}\;,\;i=1,2,3\;, \end{array}$$

where

(A3) $$\begin{array}{c} \theta =\arccos\left[\frac{2(I_{1}^{3}-9I_{1}I_{2}+27I_{3})}{2{\left[I_{1}^{2}-3I_{2}\right]}^{\frac{3}{2}}}\right]\;, \end{array}$$

taking note that since the eigenvalues $\lambda_{i}$ are distinct, we have, $I_{1}^{2}-3I_{2}\neq 0$.

The strain energy function consists of the tensors

(A4) $$\begin{array}{c} C\;,\;S_{R}\;,\;a⊗a\;. \end{array}$$

There are 18 classical invariants in the minimal integrity basis for the function (A4), i.e.,

$$\begin{array}{ccc} I_{4} & = & a\cdot Ca=\sum_{i=1}^{3}a_{i}^{2}\lambda_{i}^{2}\;,\;I_{5}=a\cdot C^{2}a=\sum_{i=1}^{3}a_{i}^{2}\lambda_{i}^{4}\;, \end{array}$$

(A5) $$\begin{array}{ccc} I_{6} & = & \mathrm{tr}\;S_{R}=\sum_{i=1}^{3}s_{ii}\;,\;I_{7}=\mathrm{tr}\;\left(S_{R}C\right)=\sum_{i=1}^{3}\lambda_{i}^{2}s_{ii}\;, \end{array}$$

(A6) $$\begin{array}{ccc} I_{8} & = & \mathrm{tr}\;\left(S_{R}C^{2}\right)=\sum_{i=1}^{3}\lambda_{i}^{4}s_{ii}\;, \end{array}$$

(A7) $$\begin{array}{ccc} I_{9} & = & \mathrm{tr}\;\left(S_{R}^{2}\right)=s_{11}^{2}+s_{22}^{2}+s_{33}^{2}+2\left(s_{12}^{2}+s_{13}^{2}+s_{23}^{2}\right)\;,\\ I_{10} & = & \mathrm{tr}\;\left(S_{R}^{2}C\right)=\lambda_{1}^{2}\left(s_{11}^{2}+s_{12}^{2}+s_{13}^{2}\right)+\\ & & \lambda_{2}^{2}\left(s_{21}^{2}+s_{22}^{2}+s_{23}^{2}\right)+\lambda_{3}^{2}\left(s_{31}^{2}+s_{32}^{2}+s_{33}^{2}\right)\;,\\ I_{11} & = & \mathrm{tr}\;\left(S_{R}^{2}C^{2}\right)=\lambda_{1}^{4}\left(s_{11}^{2}+s_{12}^{2}+s_{13}^{2}\right)+\\ & & \lambda_{2}^{4}\left(s_{21}^{2}+s_{22}^{2}+s_{23}^{2}\right)+\lambda_{3}^{4}\left(s_{31}^{2}+s_{32}^{2}+s_{33}^{2}\right)\;, \end{array}$$

(A8) $$\begin{array}{ccc} I_{12} & = & a\cdot S_{R}a\;,\;I_{13}=a\cdot S_{R}^{2}a\;,\;I_{14}=\mathrm{tr}\;A^{(2)3}\;,\\ I_{15} & = & a\cdot \left(CS_{R}a\right)\;,\;I_{16}=a\cdot \left(CS_{R}^{2}a\right)\;,\\ I_{17} & = & a\cdot \left(S_{R}C^{2}a\right)\;,\;I_{18}=a\cdot \left(C^{2}S_{R}^{2}a\right)\;. \end{array}$$

We note that the invariants $I_{\alpha }\;,\alpha =12,13,\ldots ,18$ can be explicitly expressed in terms of $\lambda_{i},a_{i}$ and $s_{ij}$ but, for brevity, we omit such explicit expressions.

From (A2) and (A5), $\lambda_{i}$ is expressed in terms of $I_{i}$. From (9) and (A5), we have 3 linear equations in $a_{i}^{2}$. On solving these linear equations we can express $a_{i}^{2}$ explicitly in terms of $I_{\alpha }\;,\alpha =1,2\ldots ,5$. The invariants $s_{ii}$ can be expressed in terms of $I_{\alpha }\;,\alpha =1,2,3,6,7,8$ by solving the 3 linear equations in (A6) for $s_{ii}$. In Equation (A7), the invariants $s_{12}^{2},s_{13}^{2},s_{23}^{2}$ appear linearly. Hence we can solve the 3 linear equations so that these invariants can be expressed in terms of $I_{\alpha }\;,\alpha =1,2,3,6,7\ldots ,11$. Since the classical invariants $I_{\alpha }\;,\alpha =12,13,\ldots ,18$ can be explicitly expressed in terms of $\lambda_{i},a_{i}$, $s_{ij}$, and taking the appropriate sign for $a_{i}$ and $s_{ij}$, they can be expressed in terms of $I_{\alpha }\;,\alpha =1,2,\ldots ,11$, indicating that only at most 11 classical invariants are independent.

## Appendix B

In this Appendix we consider a generalize strain measure, where it may contain paramaters suitable for a particular material. Consider a set of general class of Lagrangean strain tensor $\left\{F_{\left(1\right)},F_{\left(2\right)},F_{\left(3\right)}\right\}$ as defined by Hill [31]

(A9) $$\begin{array}{c} F_{\left(\alpha \right)}\left(U\right)=\sum_{i=1}^{3}f_{\left(\alpha \right)}\left(\lambda_{i}\right)u_{i}⊗u_{i}\;,\;\alpha =1,2,3\;, \end{array}$$

where $f_{\left(\alpha \right)}:\left(0,\infty \right)\to R$ is a monotonic increasing function such that

(A10) $$\begin{array}{c} f_{\left(\alpha \right)}\left(1\right)=0\;,\;f_{\left(\alpha \right)}^{\prime }\left(1\right)=1\;. \end{array}$$

In view of the above definition, we also consider $f_{\left(\alpha \right)}$ to represent physical strain measures with the extreme deformation values

(A11) $$\begin{array}{c} f_{\left(\alpha \right)}\left(\lambda_{i}\to \infty \right)=\infty \;,\;f_{\left(\alpha \right)}\left(\lambda_{i}\to 0\right)=-\infty \;. \end{array}$$

An example of a strain measure commonly used in the literature that satisfies the above properties is

(A12) $$\begin{array}{c} \ln\left(U\right)=\sum_{i=1}^{3}\ln\left(\lambda_{i}\right)u_{i}⊗u_{i}\;. \end{array}$$

Using the Lagrangean strain tensors, we construct our transversely isotropic strain energy function, as exemplified in Shariff [11], i.e.,

(A13) $$\begin{array}{ccc} W_{\left(T\right)} & = & \sum_{i=1}^{3}\left[\mu_{T}{\left(f_{\left(1\right)}\left(\lambda_{i}\right)\right)}^{2}+2\mu_{1}\alpha_{i}{\left(f_{\left(2\right)}\left(\lambda_{i}\right)\right)}^{2}\right]+\frac{\beta }{2}{\left(\sum_{i=1}^{3}\alpha_{i}f_{\left(3\right)}\left(\lambda_{i}\right)\right)}^{2}\\ & = & \mu_{T}\mathrm{tr}\;F_{\left(1\right)}^{2}+2\mu_{1}\left(F_{\left(2\right)}a\right)\cdot \left(F_{\left(2\right)}a\right)+\frac{\beta }{2}{\left(a\cdot F_{\left(3\right)}a\right)}^{2}\;. \end{array}$$

The constitutive Equation (19) can be obtained from (A13), by letting

(A14) $$\begin{array}{c} q_{1}=f_{\left(1\right)}^{2}\;,\;q_{2}=f_{\left(2\right)}^{2}\;,\;q_{3}=f_{\left(3\right)}\;. \end{array}$$

We note that, in this paper we only consider strain energy function of the form (19).

## Appendix C

Shariff [14] has discussed and proposed a model, where the fiber compression does not fully or partially contribute towards the strain energy function. He uses the functions

(A15) $$\begin{array}{c} q_{\left(p\right)}\left(x\right)=\frac{\left(1+\mathrm{erf}(a(x-1)\right)}{2}\;,\;q_{\left(n\right)}\left(x\right)=\frac{\left(1+\mathrm{erf}(a(1-x)\right)}{2}\;, \end{array}$$

where erf is the error function and *a* is a very large positive number. He then define

(A16) $$\begin{array}{c} \mu_{1}\left(I_{4}\right)=l_{p}q_{\left(p\right)}\left(I_{4}\right)+l_{n}q_{\left(n\right)}\left(I_{4}\right)\;,\;\beta \left(I_{4}\right)=m_{p}q_{\left(p\right)}\left(I_{4}\right)+m_{n}q_{\left(n\right)}\left(I_{4}\right)\;. \end{array}$$

where $l_{p}$, $l_{n}$, $m_{p}$ and $m_{n}$ are constants. Since the details of the functions in (A15) and (A16) can be found in [14], we shall not elaborate them here.

To model the fibre compression problem, the strain energy function, $W_{\left(T\right)}$ in (19) is now replaced by

(A17) $$\begin{array}{c} W_{\left(T\right)}=\mu_{T}\sum_{i=1}^{3}q_{1}\left(\lambda_{i}\right)+2\mu_{1}\left(I_{4}\right)\sum_{i=1}^{3}\alpha_{i}q_{2}\left(\lambda_{i}\right)+\frac{\beta }{2}\left(I_{4}\right){\left(\sum_{i=1}^{3}\alpha_{i}q_{3}\left(\lambda_{i}\right)\right)}^{2}\;. \end{array}$$

## Appendix D. Limitations on the Ground State Constants

Strong ellipticity condition is important in solid mechanics [32]. Restrictions on the ground state constants appearing in the strain energy function (27) are done using strong ellipticity condition at F=I. For an incompressible material, the strong ellipticity condition (see, for example, Ref. [33]) requires

(A18) m·[Q(n)m]>0,m·n=0,

where *m* and *n* are unit vectors,

(A19) $$Q\left(n\right)=Q_{1}\left(n\right)+Q_{2}\left(n\right)+Q_{3}\left(n\right)+Q_{4}\left(n\right)\;,$$

and

(A20) $$\begin{array}{ccc} Q_{1}\left(n\right) & = & \mu_{T}\left(I+n⊗n\right)\;,Q_{2}\left(n\right)=\mu_{1}\left(An⊗n+n⊗An+\left(n•An\right)I+A\right)\;, \end{array}$$

(A21) $$\begin{array}{ccc} Q_{3}\left(n\right) & = & \beta (An⊗An)\;, \end{array}$$

(A22) $$\begin{array}{ccc} Q_{4}\left(n\right) & = & \frac{3}{4}\left[\left(S_{R}n\right)\cdot n\right]I-\frac{1}{4}\left[n⊗\left(S_{R}n\right)+\left(S_{R}n\right)⊗n+S_{R}\right]\;. \end{array}$$

Following the work of Shariff and Merodio [20], we have, for plane deformations, the following inequalities:

In the case when β=0, the necessary and sufficient condition for (A18) is

(A23) $$\begin{array}{c} b_{1}>0\;\;\mathrm{and}\;b_{2}>0\;, \end{array}$$

where

(A24) $$\begin{array}{c} b_{1}=\mu_{T}+\mu_{1}\left(a_{1}^{2}+a_{2}^{2}\right)+\frac{1}{4}\left(3s_{1}-s_{2}\right)\;,\;b_{2}=\mu_{T}+\mu_{1}\left(a_{1}^{2}+a_{2}^{2}\right)+\frac{1}{4}\left(3s_{2}-s_{1}\right)\;. \end{array}$$

In the case when β≠0, (A23) and β>0 are sufficient conditions for (A18).
